# Pre-Transplant Cardiovascular Risk Factors Affect Kidney Allograft Survival: A Multi-Center Study in Korea

**DOI:** 10.1371/journal.pone.0160607

**Published:** 2016-08-08

**Authors:** Jung Nam An, Song Vogue Ahn, Jung Pyo Lee, Eunjin Bae, Eunjeong Kang, Hack-Lyoung Kim, Yong-Jin Kim, Yun Kyu Oh, Yon Su Kim, Young Hoon Kim, Chun Soo Lim

**Affiliations:** 1 Division of Nephrology, Department of Internal Medicine, Seoul National University Boramae Medical Center, Seoul, Korea; 2 Department of Critical Care Medicine, Seoul National University Boramae Medical Center, Seoul, Korea; 3 Department of Preventive Medicine, Yonsei University Wonju College of Medicine, Wonju, Gangwon-do, Korea; 4 Department of Internal Medicine, Gyeongsang National University Hospital, Changwon, Korea; 5 Division of Nephrology, Department of Internal Medicine, Seoul National University College of Medicine, Seoul, Korea; 6 Division of Cardiology, Department of Internal Medicine, Seoul National University Boramae Medical Center, Seoul, Korea; 7 Division of Cardiology, Department of Internal Medicine, Seoul National University College of Medicine, Seoul, Korea; 8 Department of Surgery, Asan Medical Center and University of Ulsan College of Medicine, Seoul, Korea; University of Edinburgh MRC Centre for Inflammation Research, UNITED KINGDOM

## Abstract

**Background:**

Pre-transplant cardiovascular (CV) risk factors affect the development of CV events even after successful kidney transplantation (KT). However, the impact of pre-transplant CV risk factors on allograft failure (GF) has not been reported.

**Methods and Findings:**

We analyzed the graft outcomes of 2,902 KT recipients who were enrolled in a multi-center cohort from 1997 to 2012. We calculated the pre-transplant CV risk scores based on the Framingham risk model using age, gender, total cholesterol level, smoking status, and history of hypertension. Vascular disease (a composite of ischemic heart disease, peripheral vascular disease, and cerebrovascular disease) was noted in 6.5% of the patients. During the median follow-up of 6.4 years, 286 (9.9%) patients had developed GF. In the multivariable-adjusted Cox proportional hazard model, pre-transplant vascular disease was associated with an increased risk of GF (HR 2.51; 95% CI 1.66–3.80). The HR for GF (comparing the highest with the lowest tertile regarding the pre-transplant CV risk scores) was 1.65 (95% CI 1.22–2.23). In the competing risk model, both pre-transplant vascular disease and CV risk score were independent risk factors for GF. Moreover, the addition of the CV risk score, the pre-transplant vascular disease, or both had a better predictability for GF compared to the traditional GF risk factors.

**Conclusions:**

In conclusion, both vascular disease and pre-transplant CV risk score were independently associated with GF in this multi-center study. Pre-transplant CV risk assessments could be useful in predicting GF in KT recipients.

## Introduction

Cardiovascular (CV) disease is a leading cause of mortality both before and after kidney transplantation (KT) [1, 2]. The occurrence of CV disease after KT is associated with sustained or accumulated CV risk factors before and after KT [3]. Pre-transplant (old age, high body mass index (BMI), and a history of CV event [4–6]) and post-transplant (new-onset hypertension or diabetes [5, 7]) CV risk factors affect the development of CV events even after successful KT. The presence of diabetes or diabetic nephropathy before KT is an independent risk factor and a strong predictor for post-transplant CV events and consequent death [8, 9]. Pre-transplant malnutrition, inflammation, and atherosclerosis are also correlated with CV outcomes after KT [10].

Specific KT-related risk factors, such as acute rejection (AR) episodes, as well as traditional CV risk factors, reportedly increase the risk of CV events after KT [11, 12]. In addition, an increased BMI after KT affects CV risk factors, including high blood pressure, an abnormal glucose profile, and an abnormal lipid profile, which leads to allograft dysfunction [13].

The associations between pre- or post-transplant CV risk factors and post-transplant CV outcomes or mortality have been extensively studied; however, whether pre-transplant CV risk factors affect kidney allograft survival has not been thoroughly investigated. Only a few research studies involving few patients or analyzing limited relationships with each risk factor have been conducted. Moreover, no studies aimed at Asian patients have been reported.

Therefore, we designed this study to assess and calculate the pre-transplant CV risk score, and to determine the association between the pre-transplant CV risk factors and kidney allograft failure (GF). We also determined the predictive performance of the pre-transplant CV risk factors for GF in Korean KT recipients.

## Methods

### Study population

Among the patients who underwent KT at Seoul National University Hospital and Asan Medical Center in Korea from January 1997 to August 2012, we enrolled a total of 2,902 patients after a thorough review of their electronic medical records. All patients were over 18 years old and had data available for our analysis. Patients who underwent either re-transplantation or combined organ transplantation and patients without information available for the analysis were excluded. This study was approved by the Institutional Review Board of Seoul National University Hospital (No. H-1409-086-609), and the requirement for informed consent was waived due to the study’s retrospective design. All clinical investigations were conducted in accordance with the guidelines of the 2013 Declaration of Helsinki.

### Data collection

Clinical parameters at the time of KT, including age, gender, BMI, smoking status, comorbidities (hypertension, diabetes mellitus, and vascular disease), the cause of end-stage renal disease (ESRD), donor factors (age, gender, and donor type), HLA mismatch, and laboratory findings (hemoglobin, serum albumin, and total cholesterol) were extracted from the electronic medical record systems of the institutions mentioned above.

Pre-transplant vascular disease was defined as a composite of ischemic heart disease, cerebrovascular accident, and peripheral vascular disease. Ischemic heart disease was diagnosed if any of the following medical histories were present: angina pectoris confirmed by coronary angiography or myocardial scintigraphy; coronary artery revascularization, such as percutaneous coronary intervention or coronary artery bypass grafting; and myocardial infarction. Cerebrovascular accident was defined as an ischemic stroke or a documented transient ischemic attack. Peripheral vascular disease included only lesions requiring revascularization.

### Definitions and classification

We calculated the pre-transplant CV risk scores based on the Framingham risk model using age, gender, history of hypertension and diabetes, smoking status, and total cholesterol level [14]. Regardless of the systolic blood pressure, two points were added to both male and female recipients with hypertension. HDL cholesterol level was excluded from the score calculation because data on HDL cholesterol was not available for all recruited patients. After the summation of each factor’s score, the calculated pre-transplant CV risk score was used as a continuous or categorical variable in the analysis. The patients were classified into three groups based on the pre-transplant CV risk score as follows: the 1^st^ tertile, 0–4 in men and 0–4 in women; the 2^nd^ tertile, 5–10 in men and 5–8 in women; the 3^rd^ tertile, ≥ 11 in men and ≥ 9 in women.

### Clinical outcomes

A researcher who was blinded to the pre-transplant CV risk score and pre-transplant clinical parameters evaluated the post-transplant clinical outcomes as follows: recurrent glomerulonephritis (GN), biopsy-proven AR, CV events, GF, and death. GF was defined as the need for permanent dialysis, allograft nephrectomy, or re-transplantation (excluding the recipient’s death despite a functioning graft). As a surrogate marker of graft failure, serum creatinine (sCr) level and estimated glomerular filtration rate (eGFR) assessed at 1, 3, 6, 9, and 12 month after KT was collected. Mortality data were collected using the electronic medical records.

### Statistical analysis

All statistical analyses were performed using SAS version 9.3 (SAS Institute, Cary, NC), R version 3.0.3 (R Development Core Team, Austria), and SPSS version 20.0 (SPSS Inc., Chicago, IL, USA) software programs. Categorical variables, which were expressed as frequencies and proportions, were compared using chi-square tests. After a test for normality, the normally distributed continuous variables were expressed as the means ± standard deviations and were compared using Student’s *t*-tests or one-way analyses of variance. The non-normally distributed variables were expressed as the medians (25^th^, 75^th^ percentiles) and were compared using the Mann-Whitney U or Kruskal-Wallis test. Cox proportional hazard models were used to calculate the hazard ratios (HRs) and 95% confidence intervals (CIs) for GF according to the baseline clinical parameters. The assumption of proportional hazards was verified using a log-minus-log plot. Biopsy-proven AR and recurrent GN were considered to be time-dependent variables. Significant covariates identified in the univariate analysis and clinically important covariates were included in the final multivariable-adjusted analysis, which was conducted in a backward stepwise manner. We also determined the HRs for GF after adjustment for competing risks of cardiac death and death of unknown causes. The impacts of the pre-transplant vascular disease or the pre-transplant CV risk score tertiles on GF were evaluated via Kaplan-Meier analysis. The cumulative incidence function was also compared according to the presence of pre-transplant vascular disease or the tertiles of the pre-transplant CV risk score using Gray’s method. The contribution of pre-transplant vascular disease or pre-transplant CV risk score for discriminating patients at high risk of GF was examined by the area under the receiver operating characteristic (ROC) curve (AUC), net reclassification improvement (NRI), and integrated discrimination improvement (IDI) [15–17]. The NRI represents the percentage change in predicted GF risk after considering the pre-transplant vascular disease or pre-transplant CV risk score in the reference model with the traditional GF risk factors. The IDI represents the sum of the average increase in sensitivity after considering the pre-transplant vascular disease or pre-transplant CV risk score among those who develop GF plus the increase in specificity after considering the pre-transplant vascular disease or pre-transplant CV risk score among those who do not develop GF. A *p* value < 0.05 was considered to be significant.

## Results

### Demographics and baseline clinical characteristics classified by the pre-transplant CV risk score

Among the total of 2,902 recruited patients, the median age was 42 years old, and 1,724 of the patients (59.4%) were male. Hypertension, diabetes, and vascular disease were observed in 84.1%, 16.6%, and 6.5% of the patients, respectively. Renal failure of unknown etiology was the most common factor necessitating KT (39.0%). The median pre-transplant CV risk score of the overall patients was 7 (4, 11). We analyzed the demographics and baseline clinical characteristics according to the pre-transplant CV risk score tertiles (Table 1). The patients in the highest tertile of pre-transplant CV risk score were more likely to have a higher BMI and pre-transplant vascular disease and had experienced KT more recently. In addition, KT from an older donor, KT from a deceased donor, HLA-DR or HLA mismatch, and the use of induction therapy for AR prevention were observed more frequently among patients in the highest tertile of pre-transplant CV risk score.

**Table 1:** Demographics and baseline clinical characteristics according to the tertiles of pre-transplant CV risk score.

		1^st^ tertile* (n = 895, 30.8%)	2^nd^ tertile* (n = 1036, 35.7%)	3^rd^ tertile* (n = 971, 33.5%)	Total (n = 2902, 100.0%)	*p*
**Male gender**		546 (61.0)	620 (59.8)	558 (57.5)	1724 (59.4)	0.280
**Age (years)**		30.0 (26.0, 34.0)	42.0 (37.3, 47.0)	53.0 (48.0, 58.0)	42.0 (33.0, 51.0)	<0.001
**Body mass index (kg/m**^**2**^**)**		20.9 (19.2, 22.9)	22.2 (20.3, 24.3)	23.1 (21.2, 25.2)	22.1 (20.1, 24.4)	<0.001
**Smoking status**	Never	795 (88.8)	799 (77.1)	680 (70.0)	2274 (78.4)	<0.001
	Former	90 (10.1)	125 (12.1)	98 (10.1)	313 (10.8)	<0.001
	Current	10 (1.1)	112 (10.8)	193 (19.9)	315 (10.9)	<0.001
**Hypertension**		669 (74.7)	860 (83.0)	912 (93.9)	2441 (84.1)	<0.001
**Diabetes mellitus**		5 (0.6)	84 (8.1)	392 (40.4)	481 (16.6)	<0.001
**Vascular disease**		10 (1.1)	44 (4.2)	135 (13.9)	189 (6.5)	<0.001
**Cause of ESRD**	GN	291 (33.9)	229 (23.1)	115 (12.5)	635 (22.9)	<0.001
	Diabetes	6 (0.7)	53 (5.3)	311 (33.7)	370 (13.3)	<0.001
	Hypertension	27 (3.1)	86 (8.7)	87 (9.4)	200 (7.2)	<0.001
	Other	169 (19.7)	178 (18.0)	138 (15.0)	485 (17.6)	<0.001
	Unknown	366 (42.6)	445 (44.9)	271 (29.4)	1082 (39.0)	<0.001
**Era**	1997–2001	292 (32.6)	291 (28.1)	189 (19.5)	772 (26.6)	<0.001
	2002–2006	262 (29.3)	312 (30.1)	266 (27.4)	840 (28.9)	<0.001
	2007–2012	341 (38.1)	433 (41.8)	516 (53.1)	1290 (44.5)	<0.001
**Age of donor (years)**		38.0 (29.0, 48.0)	39.5 (31.0, 47.0)	42.0 (30.0, 50.0)	40.0 (30.0, 48.0)	<0.001
**Male gender of donor**		515 (57.5)	585 (56.5)	563 (58.0)	1663 (57.3)	0.779
**BMI of donor (kg/m**^**2**^**)**		23.4 (21.2, 25.4)	23.4 (21.2, 25.5)	23.4 (21.4, 25.7)	23.4 (21.2, 25.6)	0.279
**Donor type**	Living related	573 (64.0)	549 (53.0)	432 (44.5)	1554 (53.5)	<0.001
	Living unrelated	146 (16.3)	281 (27.1)	279 (28.7)	706 (24.3)	<0.001
	Deceased	176 (19.7)	206 (19.9)	260 (26.8)	642 (22.1)	<0.001
**HLA-DR mismatch**	0	220 (25.4)	218 (21.7)	170 (18.2)	608 (21.7)	<0.001
	1	471 (54.4)	536 (53.3)	508 (54.3)	1515 (54.0)	<0.001
	2	175 (20.2)	251 (25.0)	257 (27.5)	683 (24.3)	<0.001
**HLA mismatch**	0–2	321 (37.3)	313 (31.3)	249 (26.9)	883 (31.7)	<0.001
	3–4	385 (44.7)	478 (47.7)	444 (48.0)	1307 (46.9)	<0.001
	5–6	155 (18.0)	210 (21.0)	232 (25.1)	597 (21.4)	<0.001
**CNIs**	Cyclosporine A	448 (54.7)	491 (51.6)	404 (45.6)	1343 (50.5)	0.004
	Tacrolimus	369 (45.1)	456 (48.2)	481 (54.3)	1309 (49.3)	0.004
**Induction therapy**		416 (46.5)	515 (49.7)	359 (63.0)	1543 (53.2)	<0.001
**Baseline laboratory findings**						
Serum albumin (mg/dL)		3.8 (3.5, 4.2)	3.7 (3.3, 4.0)	3.6 (3.2, 4.0)	3.7 (3.4, 4.1)	<0.001
Hemoglobin (g/dL)		10.3 (9.1, 11.4)	10.5 (9.3, 11.5)	10.5 (9.4, 11.6)	10.4 (9.3, 11.5)	0.003
Serum calcium (mg/dL)		9.1 (8.6, 9.6)	9.1 (8.6, 9.7)	9.1 (8.6, 9.7)	9.1 (8.6, 9.7)	0.936
Serum phosphorus (mg/dL)		5.4 (4.4, 6.6)	5.2 (4.2, 6.3)	4.9 (3.9, 5.9)	5.1 (4.1, 6.2)	<0.001
Serum total cholesterol (mg/dL)		148 (128, 170)	163 (138, 188)	172 (144, 203)	160.0 (136.0, 187.0)	<0.001
Serum glucose (mg/dL)		100.0 (85.0, 116.0)	108.0 (90.0, 128.0)	116.5 (93.0, 153.8)	106.0 (89.0, 129.0)	<0.001
Serum creatinine (mg/dL)		9.6 (7.3, 12.6)	9.1 (6.9, 11.5)	8.1 (6.1, 10.4)	8.8 (6.7, 11.4)	<0.001

Data are presented as the median (25^th^, 75^th^ percentiles) or as a number (percent).

* 1^st^ tertile, 0–4 in men and 0–4 in women; 2^nd^ tertile, 5–10 in men and 5–8 in women; 3^rd^ tertile, ≥ 11 in men and ≥ 9 in women

BMI, body mass index; CNIs, calcineurin inhibitors; ESRD, end-stage renal disease; GN, glomerulonephritis

### Post-transplant outcomes and comparisons according to the allograft failure

During the median follow-up of 6.4 (0.0–17.8) years after KT, 286 patients (9.9%) had experienced GF and 122 (4.2%) patients had died. The causes of death are listed in S1 Table. Death of unknown etiology was the most common cause (32.0%), followed by infections (22.1%), and respiratory (19.7%) and cardiac (11.5%) causes.

Patients who experienced GF were more likely to be male, current smokers and received a kidney from a deceased donor (Table 2). Much more frequent HLA-DR mismatch and lower pre-transplant hemoglobin concentrations were found among GF patients. The use of tacrolimus as an immunosuppression and the use of induction therapy for AR prevention were more frequent in the patients without GF. However, age; comorbidities, including hypertension, diabetes, and vascular disease; donor age; and donor gender were not significantly different between the patients with and without GF.

**Table 2:** Demographics and baseline clinical characteristics according to the development of allograft failure.

		Allograft failure (n = 286, 9.9%)	No allograft failure (n = 2616, 90.1%)	Total (n = 2902, 100.0%)	*p*
**Male gender**		190 (66.4)	1534 (58.6)	1724 (59.4)	0.011
**Age (years)**		41.5 (32.0, 49.3)	42.0 (33.0, 50.8)	42.0 (33.0, 51.0)	0.444
**Body mass index (kg/m**^**2**^**)**		22.7 (20.5, 24.5)	22.0 (20.1, 24.3)	22.1 (20.1, 24.4)	0.042
**Smoking status**	Never	220 (76.9)	2054 (78.5)	2274 (78.4)	<0.001
	Former	18 (6.3)	295 (11.3)	313 (10.8)	<0.001
	Current	48 (16.8)	267 (10.2)	315 (10.9)	<0.001
**Hypertension**		242 (84.6)	2199 (84.1)	2441 (84.1)	0.807
**Diabetes mellitus**		52 (18.2)	429 (16.4)	481 (16.6)	0.441
**Vascular disease**		26 (9.1)	163 (6.2)	189 (6.5)	0.063
**Cause of ESRD**	GN	50 (17.9)	585 (23.5)	635 (22.9)	<0.001
	Diabetes	40 (14.3)	330 (13.2)	370 (13.3)	<0.001
	Hypertension	11 (3.9)	189 (7.6)	200 (7.2)	<0.001
	Other	35 (12.2)	450 (17.2)	485 (16.7)	<0.001
	Unknown	144 (51.4)	938 (37.6)	1082 (39.0)	<0.001
**Era**	1997–2001	171 (59.8)	601 (23.0)	772 (26.6)	<0.001
	2002–2006	78 (27.3)	762 (29.1)	840 (28.9)	<0.001
	2007–2012	37 (12.9)	1253 (47.9)	1290 (44.5)	<0.001
**Age of donor (years)**		40.5 (29.0, 47.0)	40.0 (30.0, 48.0)	40.0 (30.0, 48.0)	0.480
**Male gender of donor**		163 (57.0)	1500 (57.3)	1663 (57.3)	0.910
**BMI of donor (kg/m**^**2**^**)**		23.2 (20.8, 25.2)	23.4 (21.3, 25.6)	23.4 (21.2, 25.6)	0.177
**Donor type**	Living related	122 (42.7)	1432 (54.7)	1554 (53.5)	<0.001
	Living unrelated	79 (27.6)	627 (24.0)	706 (24.3)	<0.001
	Deceased	85 (29.7)	557 (21.3)	642 (22.1)	<0.001
**HLA-DR mismatch**	0	43 (15.8)	565 (22.3)	608 (21.7)	0.039
	1	162 (59.6)	1353 (53.4)	1515 (54.0)	0.039
	2	67 (24.6)	616 (24.3)	683 (24.3)	0.039
**HLA mismatch**	0–2	68 (25.0)	815 (32.4)	883 (31.7)	0.040
	3–4	143 (52.6)	1164 (46.3)	1307 (46.9)	0.040
	5–6	61 (22.4)	536 (21.3)	597 (21.4)	0.040
**CNIs**	Cyclosporine A	178 (62.2)	1165 (44.5)	1343 (50.5)	<0.001
	Tacrolimus	102 (35.7)	1207 (46.1)	1309 (49.3)	<0.001
**Induction therapy**		77 (26.9)	1466 (56.0)	1543 (53.2)	<0.001
**Baseline laboratory findings**					
Serum albumin (mg/dL)		3.7 (3.4, 4.1)	3.7 (3.3, 4.1)	3.7 (3.4, 4.1)	0.165
Hemoglobin (g/dL)		10.0 (8.7, 10.9)	10.5 (9.3, 11.6)	10.4 (9.3, 11.5)	<0.001
Serum calcium (mg/dL)		9.0 (8.5, 9.7)	9.1 (8.6, 9.6)	9.1 (8.6, 9.7)	0.339
Serum phosphorus (mg/dL)		5.3 (4.3, 6.5)	5.1 (4.1, 6.2)	5.1 (4.1, 6.2)	0.096
Serum total cholesterol (mg/dL)		162.0 (139.0, 185.5)	160.0 (135.0, 187.8)	160.0 (136.0, 187.0)	0.391
Serum glucose (mg/dL)		104.0 (87.0, 128.8)	107.0 (90.0, 130.0)	106.0 (89.0, 129.0)	0.099
Serum creatinine (mg/dL)		9.8 (7.6, 13.0)	8.8 (6.7, 11.2)	8.8 (6.7, 11.4)	<0.001

Data are presented as the median (25^th^, 75^th^ percentiles) or as a number (percent).

BMI, body mass index; CNIs, calcineurin inhibitors; ESRD, end-stage renal disease; GN, glomerulonephritis

### Pre-transplant cardiovascular risk and allograft failure

**Fig 1:**
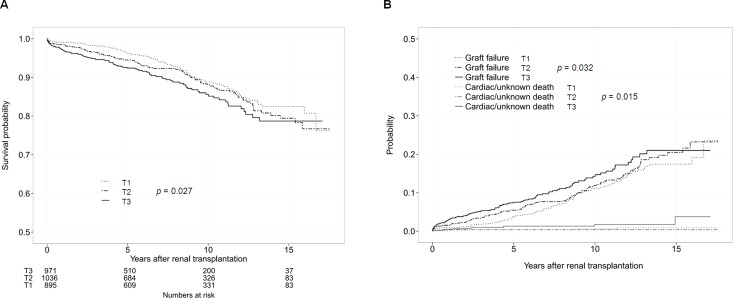
Allograft failure according to the pre-transplant CV risk score before and after adjustment for competing risks. (A) Kaplan-Meier curves for the probability of graft failure in the KT recipients classified by the pre-transplant CV risk score tertiles. (B) Cumulative incidence function for graft failure and competing risk by the pre-transplant CV risk score tertiles. Cardiac death and unknown death were considered competing risk events. T1, the 1^st^ tertile; T2, the 2^nd^ tertile; T3, the 3^rd^ tertile.

**Fig 2:**
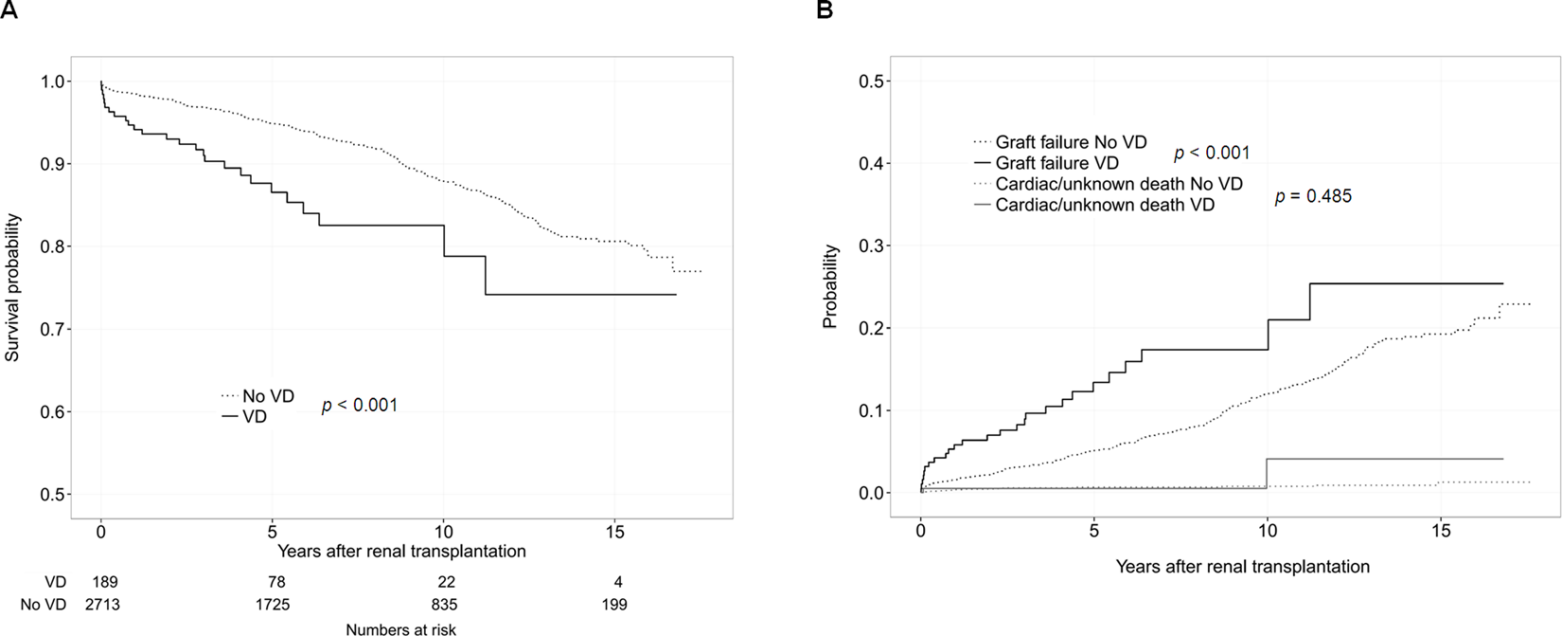
Allograft failure according to the pre-transplant vascular disease before and after adjustment for competing risks. (A) Kaplan-Meier curves for the probability of graft failure in the KT recipients classified by the presence of pre-transplant vascular disease. (B) Cumulative incidence function for graft failure and competing risk by the presence of pre-transplant vascular disease. Cardiac death and unknown death were considered competing risk events. VD, vascular disease.

To investigate the association between the pre-transplant CV risk and GF, we performed a Cox proportional hazard regression analysis and Kaplan-Meier survival analysis (Table 3, Figs 1A and 2A). In the multivariable-adjusted models, the pre-transplant CV risk score and the presence of pre-transplant vascular disease were associated with an increased risk of GF (Models 1, 2, and 3). Even after adjustment for vascular disease, the HR for GF (comparing the highest with the lowest tertile of pre-transplant CV risk score) was 1.49 (95% CI 1.09–2.04) (Model 4), and the risk for GF increased by 3% when pre-transplant CV risk score increased by 1 (Model 5). Moreover, the patients with pre-transplant vascular disease had an approximately two-fold higher risk of GF. Significantly higher sCr levels and lower eGFR concentrations were also observed in the KT recipients with pre-transplant vascular disease and with the highest tertile of pre-transplant CV risk score (S2 and S3 Tables).

**Table 3:** Hazard ratios for allograft failure according to baseline risk factors in 2,902 subjects.

		Model 1*		Model 2*		Model 3*		Model 4*		Model 5*	
		HR (95% CI)	*p*	HR (95% CI)	*p*	HR (95% CI)	*p*	HR (95% CI)	*p*	HR (95% CI)	*p*
**Pre-transplant CV Risk Score**^**§**^	1^st^ tertile	1.00		-		-		1.00		-	
	2^nd^ tertile	1.19 (0.89–1.60)	0.237	-		-		1.17 (0.87–1.58)	0.286	-	
	3^rd^ tertile	1.65 (1.22–2.23)	0.001	-		-		1.49 (1.09–2.04)	0.014	-	
**Pre-transplant CV Risk Score (per 1 score increase)**		-		1.05 (1.02–1.07)	0.001	-		-		1.03 (1.01–1.06)	0.023
**Vascular disease**	No	-		-		1.00		1.00		1.00	
	Yes	-		-		2.51 (1.66–3.80)	<0.001	2.17 (1.41–3.34)	<0.001	2.13 (1.37–3.30)	<0.001
**Recipient gender**	Male	1.00		1.00		1.00		1.00		1.00	
	Female	0.90 (0.70–1.16)	0.421	0.96 (0.75–1.24)	0.780	0.93 (0.72–1.20)	0.572	0.92 (0.72–1.19)	0.527	0.97 (0.75–1.25)	0.796
**Induction therapy**	No	1.00		1.00		1.00		1.00		1.00	
	Yes	0.53 (0.40–0.71)	<0.001	0.53 (0.40–0.71)	<0.001	0.53 (0.40–0.71)	<0.001	0.52 (0.39–0.70)	<0.001	0.53 (0.39–0.70)	<0.001
**Recurrent glomerulonephritis**^**†**^	No	1.00		1.00		1.00		1.00		1.00	
	Yes	6.62 (5.05–8.67)	<0.001	6.53 (4.99–8.54)	<0.001	6.50 (4.97–8.50)	<0.001	6.78 (5.17–8.88)	<0.001	6.68 (5.11–8.75)	<0.001
**Biopsy-proven acute rejection**^**†**^	No	1.00		1.00		1.00		1.00		1.00	
	Yes	6.48 (5.05–8.31)	<0.001	6.50 (5.06–8.34)	<0.001	6.54 (5.10–8.39)	<0.001	6.48 (5.05–8.31)	<0.001	6.49 (5.06–8.33)	<0.001
**Age of donor (per 1 year increase)**		1.00 (0.99–1.01)	0.614	1.00 (0.99–1.01)	0.621	1.01 (0.99–1.02)	0.367	1.00 (0.99–1.01)	0.541	1.00 (0.99–1.01)	0.529
**Donor type**	Living related	1.00		1.00		1.00		1.00		1.00	
	Living unrelated	2.11 (1.57–2.83)	<0.001	2.05 (1.53–2.76)	<0.001	2.05 (1.53–2.76)	<0.001	2.02 (1.50–2.72)	<0.001	1.99 (1.48–2.68)	<0.001
	Deceased donor	1.92 (1.45–2.55)	<0.001	1.91 (1.44–2.54)	<0.001	1.94 (1.46–2.57)	<0.001	1.93 (1.45–2.56)	<0.001	1.92 (1.45–2.56)	<0.001

*Model 1: pre-transplant CV risk score by tertile; Model 2: pre-transplant CV risk score as a continuous variable; Model 3: pre-transplant vascular disease; Model 4: pre-transplant CV risk score tertiles plus pre-transplant vascular disease; Model 5: continuous pre-transplant CV risk score plus pre-transplant vascular disease; all adjusted for traditional risk factors (traditional risk factors of GF include recipient gender, induction therapy, donor age, donor type, recurrent glomerulonephritis, and biopsy-proven acute rejection)

^§^1^st^ tertile, 0–4 in men and 0–4 in women; 2^nd^ tertile, 5–10 in men and 5–8 in women; 3^rd^ tertile, ≥ 11 in men and ≥ 9 in women

^†^ used as time-dependent variables

### Hazard ratios for allograft failure after adjustment for competing risks

Next, we determined the HRs for GF after adjustment for competing risks of death. First, in the risk model considering the competing risk of cardiac death, the pre-transplant CV risk score and the presence of pre-transplant vascular disease significantly increased the risk for GF (Model 1, the highest tertile HR 1.54, 95% CI 1.10–2.15; Model 2, HR 1.04, 95% CI 1.01–1.07; Model 3, HR 2.05, 95% CI 1.25–3.38) (Table 4). Second, after adjustment for competing risks of cardiac and unknown death, the pre-transplant vascular disease was an independent risk factor for GF (increasing the risk by 2.19 times). The pre-transplant CV risk score was also associated with an increased risk of GF; the HR for GF (comparing the highest with the lowest tertile of pre-transplant CV risk score) was 1.46 (95% CI 1.09–2.04), and the risk for GF increased by 3% when pre-transplant CV risk score increased by 1.

**Table 4:** Hazard ratios for allograft failure with adjustment for competing risks in 2,902 subjects.

		Without competing risk		With competing risk of cardiac death		With competing risks of cardiac & unknown death	
		HR (95% CI)	*p*	HR (95% CI)	*p*	HR (95% CI)	*p*
Model 1*							
Pre-transplant CV Risk Score^§^	1^st^ tertile	1.00		1.00		1.00	
	2^nd^ tertile	1.19 (0.89–1.60)	0.237	1.16 (0.84–1.59)	0.370	1.17 (0.86–1.61)	0.320
	3^rd^ tertile	1.65 (1.22–2.23)	0.001	1.54 (1.10–2.15)	0.012	1.46 (1.05–2.03)	0.025
Model 2*							
Pre-transplant CV Risk Score (per 1 score increase)		1.05 (1.02–1.07)	0.001	1.04 (1.01–1.07)	0.026	1.03 (1.01–1.06)	0.029
Model 3*							
Vascular disease		2.51 (1.66–3.80)	<0.001	2.05 (1.25–3.38)	0.005	2.19 (1.35–3.55)	0.002

* In models 1–3, adjustments for recipient gender, induction therapy, donor age, donor type, recurrent glomerulonephritis, and biopsy-proven acute rejection were performed.

^§^ 1^st^ tertile, 0–4 in men and 0–4 in women; 2^nd^ tertile, 5–10 in men and 5–8 in women; 3^rd^ tertile, ≥ 11 in men and ≥ 9 in women

In cumulative incidence function test after adjustment for competing risks of cardiac and unknown death (Figs 1B and 2B), the pre-transplant CV risk score and the presence of pre-transplant vascular disease were associated with an increased risk of GF.

We examined whether the addition of the pre-transplant CV risk score or vascular disease could better predict GF compared to the traditional GF risk factors, including recipient gender, induction therapy, donor age, donor type, recurrent GN, and biopsy-proven AR (Table 5). In the ROC analysis, the addition of pre-transplant CV risk score, pre-transplant vascular disease, or both did not increase the AUC for the prediction of GF. However, an analysis using the NRI showed that the inclusion of pre-transplant CV risk score, vascular disease, and the combination of both factors significantly improved the net risk reclassification by 14.2% (95% CI 5.5–22.8%; *p* = 0.001), 9.7% (95% CI 4.2–15.1%; *p* = 0.028), and 12.8% (95% CI 4.2–21.4%; *p* = 0.004), respectively, compared to the traditional GF risk factors. Furthermore, the inclusion of pre-transplant CV risk score, vascular disease, and both exhibited modest but significant increases in the IDI (pre-transplant CV risk score 0.003 [95% CI 0.001–0.006], *p* = 0.021; vascular disease 0.003 [95% CI 0.001–0.005], *p* = 0.006; both pre-transplant CV risk score and vascular disease 0.005 [95% CI 0.001–0.008]; *p* = 0.005).

**Table 5:** Incremental value of pre-transplant CV risk score or vascular disease over traditional risk factors for predicting allograft failure.

	ROC analysis		NRI analysis		IDI analysis	
	AUC (95% CI)	*p*	NRI (95% CI)	*p*	IDI (95% CI)	*p*
Model 1 (Traditional risk factors*)	0.789 (0.762–0.815)		reference		reference	
**Model 1 + Pre-transplant CV Risk Score**	0.791 (0.765–0.817)	0.405	0.142 (0.055–0.228)	0.001	0.003 (0.001–0.006)	0.021
**Model 1 + Vascular disease**	0.790 (0.764–0.817)	0.576	0.097 (0.042–0.151)	0.028	0.0028 (0.001–0.005)	0.006
**Model 1 + Pre-transplant CV Risk Score+ Vascular disease**	0.790 (0.764–0.817)	0.576	0.128 (0.042–0.214)	0.004	0.0046 (0.001–0.008)	0.005

* Traditional risk factors of GF include recipient gender, induction therapy, donor age, donor type, recurrent glomerulonephritis, and biopsy-proven acute rejection. AUC, area under the curve; CI, confidence interval; IDI, integrated discrimination improvement; NRI, net reclassification improvement; ROC, receiver operating characteristic

## Discussion

In this study, a higher CV risk score and the presence of vascular disease before KT were independently associated with GF after adjustments for the competing risks of cardiac and unknown death. The addition of the pre-transplant CV risk score or the presence of vascular disease to traditional GF risk factors significantly improved the discrimination power for GF prediction.

Recently, it was reported that measured CV risk factors did not affect KT outcomes [18] and that the pre-transplant CV risk was not associated with death-censored graft survival [19]. However, these studies included a relatively small number of KT recipients. Although Sung et al. [20] found that a smoking history before KT increased the risk of GF more than two-fold and Kheradmand et al. [21] reported that allograft loss was caused by pre-transplant smoking, these studies were also conducted on relatively few patients in a single center and analyzed only the smoking status among the various pre-transplant CV risk factors.

To perform a more comprehensive analysis, we calculated the CV risk score using various pre-transplant CV risk factors in this study. The median value of pre-transplant CV risk score in our study population was low, and the assumed CV disease risk was also relatively low [14]. In fact, a low cumulative incidence of post-transplant CV events (2.8%) was observed. The observation may be due to the patient ethnicity and the lower prevalence of diabetes and/or vascular diseases compared to previous studies [2].

The recipients with a higher pre-transplant CV risk score tended to have several GF-related factors, such as having undergone KT from a deceased donor or older donor, and were more likely to have an HLA-DR or HLA mismatch and to have undergone KT recently. These findings suggest that the KT candidates have been increasingly expanded to include patients with higher risk for KT-related complications, CV events, or mortality. Additionally, these findings raise the question of whether the association between pre-transplant CV risk score and GF was attributable to these confounding variables. However, we demonstrated the definite effects of pre-transplant CV score on GF through a multivariable analysis that was adjusted for the aforementioned factors and for the established GF risk factors, including biopsy-proven AR and recurrent GN. Although pre-transplant vascular disease was closely related to pre-transplant CV risk score, simultaneous adjustment for these two factors still showed that these factors were independently associated with GF.

Some pre-transplant CV risk factors are known to contribute to early post-transplant mortality [8, 9, 22, 23]. Thus KT recipients with a high CV risk may have an altered GF probability. Therefore, we adjusted for the competing risks of cardiac death and death due to unknown causes, which were most likely to be affected by the pre-transplant CV risk. The results revealed that both the pre-transplant CV risk score and the vascular disease remained independently associated with GF. Furthermore, these factors improved the predictability of GF compared to traditional GF risk factors. These findings seemed to have relevance to those of our recent study, indicating that post-transplant CV risk factors such as persistent left ventricular hypertrophy and high systolic blood pressure adversely affected allograft survival [24].

Despite the fact that the mechanisms by which pre-transplant CV risk affects GF are still uncertain, it is encouraging that identifiable and modifiable pre-transplant risk factors for GF were revealed in a large Asian cohort study with a relatively long follow-up period. Our results indicate the necessity of identifying and managing CV risk factors in KT candidates.

However, our study had a few limitations that should be noted. First, the causes of all deaths after KT were not clear. This might have affected the results of the competing risk analysis with death due to unknown causes. Second, data for all pre-transplant CV risk score components were not available; however, we analyzed the data in various ways and demonstrated the clinical impact of the modified pre-transplant CV risk score on GF occurrence. Third, the number of patients with unknown cause of ESRD was not inconsiderable; however, this number was not different depending on the era, and besides, the poorer long-term outcomes were not seen in the patients with the unknown cause of ESRD compared to those with the other causes of ESRD [25]. Naturally, further research regarding the associations between pre-transplant CV risk and GF and whether CV risk reduction before KT can reduce the risk for GF should be performed via well-designed, prospective, large cohort studies.

In conclusion, both the pre-transplant CV risk score and the presence of pre-transplant vascular diseases are independently associated with GF even after adjustment for the competing risks of cardiac and unknown death. Pre-transplant CV risk assessments could be useful to predict GF risk. Consequently, identification of KT candidates with high CV risk and careful monitoring of the CV risk factors may facilitate GF detection in KT recipients.

## Supporting Information

S1 Table The clinical outcomes after kidney transplantation. (DOCX)

S2 Table Post-transplant renal function according to the presence of pre-transplant vascular disease. (DOCX)

S3 Table Post-transplant renal function according to the pre-transplant CV risk score tertiles. (DOCX)

## Abbreviations

AR: acute rejection
AUC: area under the curve
CV: cardiovascular
ESRD: end-stage renal disease
GF: allograft failure
GN: glomerulonephritis
IDI: integrated discrimination improvement
KT: kidney transplantation
NRI: net reclassification improvement
ROC: receiver operating characteristic
