# Supplementary material for: Pre-Transplant Cardiovascular Risk Factors Affect Kidney Allograft Survival: A Multi-Center Study in Korea
Source: PLoS One. 2016 Aug 8;11(8):e0160607. doi: 10.1371/journal.pone.0160607 (PMC4976895; doi:10.1371/journal.pone.0160607)
Supplement: S1 Table — (DOCX) [file pone.0160607.s001.docx]

**S1 Table. The clinical outcomes after kidney transplantation**

|  | | **Total**  **(n=2902, 100.0%)** |
| --- | --- | --- |
| **Post-transplant diabetes mellitus** | | 268 (9.2) |
| **Recurrent glomerulonephritis** | | 219 (7.5) |
| **Biopsy-proven acute rejection** | | 664 (22.9) |
| **Post-transplant cardiovascular event** | | 80 (2.8) |
| **Death** | | 122 (4.2) |
| **Cause of death** | Infection | 27 (22.1) |
|  | Respiratory | 24 (19.7) |
|  | Cardiac | 14 (11.5) |
|  | Cancer | 11 (9.0) |
|  | Unknown | 39 (32.0) |
|  | Others | 7 (5.7) |

Data are presented as a number (percent).
