# Supplementary material for: Pre-Transplant Cardiovascular Risk Factors Affect Kidney Allograft Survival: A Multi-Center Study in Korea
Source: PLoS One. 2016 Aug 8;11(8):e0160607. doi: 10.1371/journal.pone.0160607 (PMC4976895; doi:10.1371/journal.pone.0160607)
Supplement: S2 Table — (DOCX) [file pone.0160607.s002.docx]

**S2 Table. Post-transplant renal function according to the presence of pre-transplant vascular disease**

|  | | **Vascular disease (+)**  **(n=189, 6.5%)** | **Vascular disease (-)**  **(n=2713, 93.5%)** | ***p*** |
| --- | --- | --- | --- | --- |
| **Serum creatinine (mg/dL)** | |  |  |  |
|  | **After 1 month** | 1.3 (1.0, 1.5) | 1.2 (1.0, 1.4) | 0.006 |
|  | **After 3 month** | 1.2 (1.0, 1.4) | 1.1 (0.9, 1.4) | <0.001 |
|  | **After 6 month** | 1.3 (1.1, 1.4) | 1.2 (1.0, 1.4) | 0.030 |
|  | **After 9 month** | 1.2 (1.1, 1.4) | 1.2 (1.0, 1.4) | 0.006 |
|  | **After 12 month** | 1.2 (1.1, 1.5) | 1.2 (1.0, 1.4) | 0.003 |
| **Estimated GFR (mL/min/1.73 m^2^)** | | |  |  |
|  | **After 1 month** | 62.5 (51.8, 73.2) | 65.9 (54.7, 78.3) | 0.011 |
|  | **After 3 month** | 62.4 (51.6, 76.7) | 68.7 (55.8, 84.2) | <0.001 |
|  | **After 6 month** | 62.1 (51.9, 73.2) | 63.9 (54.1, 75.4) | 0.090 |
|  | **After 9 month** | 62.5 (53.5, 71.3) | 65.1 (55.2, 76.9) | 0.017 |
|  | **After 12 month** | 62.5 (51.6, 72.4) | 65.0 (54.7, 76.4) | 0.009 |

Data are presented as the median (25^th^, 75^th^ percentiles).
