# Supplementary material for: Pre-Transplant Cardiovascular Risk Factors Affect Kidney Allograft Survival: A Multi-Center Study in Korea
Source: PLoS One. 2016 Aug 8;11(8):e0160607. doi: 10.1371/journal.pone.0160607 (PMC4976895; doi:10.1371/journal.pone.0160607)
Supplement: S3 Table — (DOCX) [file pone.0160607.s003.docx]

**S3 Table. Post-transplant renal function according to the pre-transplant CV risk score tertiles**

|  | | **1^st^ tertile^*^**  **(n=895, 30.8%)** | | **2^nd^ tertile^*^**  **(n=1036, 35.7%)** | | **3^rd^ tertile^*^**  **(n=971, 33.5%)** | ***p*** |
| --- | --- | --- | --- | --- | --- | --- | --- |
| **Serum creatinine (mg/dL)** | |  | |  | |  |  |
|  | **After 1 month** | 1.2 (1.0, 1.4) | | 1.2 (1.0, 1.4) | | 1.2 (1.0, 1.4) | 0.028 |
|  | **After 3 month** | 1.1 (0.9, 1.4) | | 1.2 (0.9, 1.4) | | 1.2 (0.9, 1.4) | 0.798 |
|  | **After 6 month** | 1.2 (1.0, 1.4) | | 1.2 (1.0, 1.4) | | 1.2 (1.0, 1.4) | 0.002 |
|  | **After 9 month** | 1.2 (1.0, 1.4) | | 1.2 (1.0, 1.4) | | 1.2 (1.0, 1.4) | 0.008 |
|  | **After 12 month** | 1.2 (1.0, 1.4) | | 1.2 (1.0, 1.4) | | 1.2 (1.0, 1.4) | <0.001 |
| **Estimated GFR (mL/min/1.73 m^2^)** | | |  | |  | |  |
|  | **After 1 month** | 68.8 (57.7, 80.6) | | 65.1 (55.0, 77.4) | | 62.5 (51.3, 76.2) | <0.001 |
|  | **After 3 month** | 73.8 (59.8, 89.1) | | 68.5 (55.8, 82.0) | | 64.1 (51.8, 80.2) | <0.001 |
|  | **After 6 month** | 66.8 (56.8, 78.3) | | 63.8 (53.9, 75.4) | | 61.2 (51.5, 73.1) | <0.001 |
|  | **After 9 month** | 67.6 (57.7, 79.4) | | 64.4 (55.3, 76.0) | | 62.2 (52.4, 73.7) | <0.001 |
|  | **After 12 month** | 67.2 (56.7, 78.5) | | 64.4 (54.1, 75.4) | | 62.4 (52.7, 74.5) | <0.001 |

Data are presented as the median (25^th^, 75^th^ percentiles).

^*^ 1^st^ tertile, 0-4 in men and 0-4 in women; 2^nd^ tertile, 5-10 in men and 5-8 in women; 3^rd^ tertile, ≥ 11 in men and ≥ 9 in women
